# Identification of geographical origins of *Panax notoginseng* based on HPLC multi-wavelength fusion profiling combined with average linear quantitative fingerprint method

**DOI:** 10.1038/s41598-021-84589-9

**Published:** 2021-03-04

**Authors:** Jing Bai, Pan Yue, Qiang Dong, Fang Wang, Chengyan He, Yang Li, Jinlin Guo

**Affiliations:** Development and Utilization of Chinese Medicine Resources Key Laboratory Breeding Base, Key Laboratory of Systematic Research, The Ministry of Education Key Laboratory of Standardization of Chinese Herbal Medicine, Chengdu University of Traditional Chinese Medicine, Chengdu, 611137 China

**Keywords:** Drug discovery, Chemistry

## Abstract

The aim of this study was to establish a method for geographical origins identification of *Panax notoginseng* (*P. notoginseng*) based on abundant chromatographic spectral information. Characteristic fingerprints of *P. notoginseng* extracts samples were generated by Multi-wavelength Fusion Profiling (MWFP) method based on the HPLC fingerprints established at three wavelengths of 203 nm, 270 nm and 325 nm. The samples grouping results calculated with the averagely linear quantified fingerprint method (ALQFM) and the unsupervised statistical methods based on fusion fingerprints matches with the geographical origins. The Multi-wavelength Fusion Profiling (MWFP) method has been successfully applied to identification of geographical origins of *P. notoginseng* and shows the advantages compared with single—channel fingerprints. In addition, eight physiologically active components, including four saponins, two flavones and two amino acids, were identified from the most relevant ingredients of *P. notoginseng* geographical origins by fusion fingerprint-efficacy relationship analysis. Besides the recognized active saponins, other categories of active ingredients such as flavonoids and amino acids should be paid attention to in the producing areas identification or the quality judgment of *P. notoginseng*.

## Introduction

*Panax notoginseng* named “San-qi” and “Tian-qi” is the dried roots and rhizomes of *Panax notogitiseng* (Burk.) F. H. Chen listed in the Chinese pharmacopoeia. It is a virtual Chinese medicine (TCM) with an annual output value exceeded 70 billion RMB yuan and more than 3600 related drug approval numbers in China^1^. Its main efficacy is promoting blood circulation, removing blood stasis, detumescence and analgesia^2–4^.

The genuine producing area of *P. notoginseng* was in Yunnan province, China^5^. Due to the great demand cannot be met, the production of *P. notoginseng* expanded to the adjacent region. Suining City, Sichuan Province was the first to introduce successfully, and the only production area outside Yunnan with stable output for three consecutive years up to 2019. The difference of producing area involves the distinction in complicate natural and artificial factors, which will lead to the diversity of the integral composition and eventually the efficacy of Traditional Chinese Medicine^6,7^. In terms of geographical conditions, the *P. notoginseng* origin in Yunnan is significantly different from that in Sichuan. The former is plateau mountain area and the latter is hills in the hinterland of the basin. The natural environment and the growing techniques are both various in the two provinces producing area. Therefore, it is necessary to distinguish *P. notoginseng* produced in the two provinces by origin discriminant technique. Basing on the current legal quality indicators of *P. notoginseng* regulated in Pharmacopoeia of China, the individual indicators of Ginsenoside Rb_1_, Ginsenoside Rg_1_ and Notoginsenoside R_1_ content, the identification of geographical origins is difficult to achieve. There were some studies of the identification of geographical origins of TCM^8,9^ including *P. notoginseng*^10^ based on stable isotope ratios. Except the strategy related to the inorganic stable isotope the origins identified strategy based on complex organic composition information have been worked out. *P. notoginseng* geographical discrimination models have been established based on spectral data and various data mining algorithms. In Wang’s study^11^, the model is established by partial least squares discriminant analysis (PLS-DA) of the optimized UV spectra data of *P. notoginseng* samples. The Fourier transform-infrared spectrum^12^ or near-infrared spectrum data of *P. notoginseng* are used to distinguish the producing area^13^. In order to exploit the synergetic and complementary information, researchers established models of origin discrimination based on high-level fusion of Fourier transform mid-infrared spectroscopy and near infrared spectroscopy combined with the algorithm of random forest^14,15^. Different from the methods based on spectral data matrix mentioned above, the discrimination model based on near-infrared spectra image and deep learning strategy is also applied to *P. notoginseng*^16^. These discrimination methods based on spectral analysis have an advantage in applying the spectral information in a certain wavelength range of samples comprehensively. However, the information of spectral without separation is complex and could only be used for discriminant analysis after calculation and transformation. At the same time, it is difficult to directly associate the information of components with the spectral and established discriminant model.

At present, researchers discussed Spectral and Chromatographic Overall Analysis technique (SCOA), aiming to extract, integrate and transform the information from complex multi-channel into analyzable forms^17^. Multi-wavelength Fusion Profiling (MWFP) is a representative of the SCOA. It is obtained by the projection along the wavelength axis of the HPLC chromatograms collected from the same sample analyzed with multiple wavelengths (or DAD). It is chromatogram maximizes the available Multi-wavelength chromatographic information. MWFP could provide more information for origin identification than single wavelength HPLC fingerprint, and the more accurate conclusion may be obtained with MWFP analysis. Compared to the spectral without separation, chromatographic separation reduces the complexity of the spectrum, and the relationship between chromatographic peaks and chemical compositions is more direct. In recent studies, MWFP method was applied for the quality assessment of the single herbs^18^ and proprietary Chinese medicine with more complex components consisting of a variety of medicines^19^. These studies based on the MWFP method provide novel and comprehensive strategy for TCM quality assessment.

In this present study, a Multi-wavelength Fusion Profiling (MWFP) method is developed to distinguish the *P. notoginseng* samples from two producing area with various geographical conditions.

## Materials and methods

### Chemical and materials

**Figure 1:**
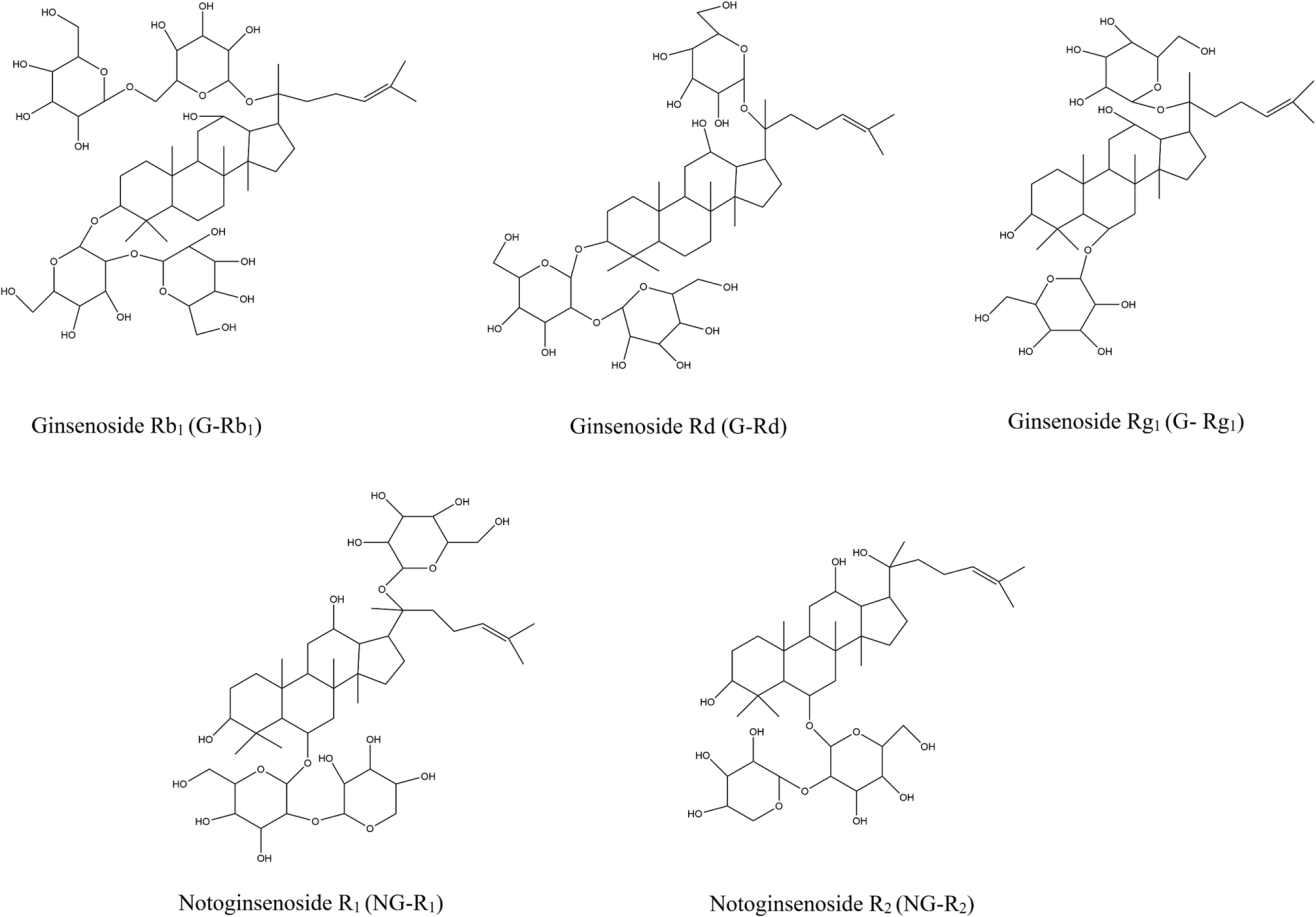
Chemical structures of five reference standards.

Acetonitrile and methanol (HPLC-grade) were obtained from Sigma-Aldrich (Steinheim, Germen). Formic acid (HPLC-grade) was supplied from the local dealer. Ultra-pure water was made by the pure water machine (Millipore, France, 18.2 MΩcm@25 °C). Reference standards of Ginsenoside Rb_1_ (G-Rb_1_), Ginsenoside Rg_1_ (G-Rg_1_), Ginsenoside Rd (G-Rd), Notoginsenoside R_1_ (NG-R_1_), Notoginsenoside R_2_ (NG-R_2_) were acquired from Chengdu Glip Biotechnology Co., Ltd., Chengdu, Sichuan. The structures of the five saponins were presented in Fig. 1.

### Sample sources

The *P. notoginseng* samples S1–S15 were collected in Suining City, Sichuan Province; S16–S19 and S23–S26 were collected in Wenshan Zhuang and Miao Autonomous Prefecture, Yunnan Province, S20–S22 were collected in Zhaotong, Yunnan Province, and S27–S32 were collected in Hani-Yi Autonomous Prefecture of Honghe, Yunnan Province, which are the main producing areas of *P. notoginseng*.

### Sample preparation and HPLC analysis

The *P. notoginseng* samples were smashed by the pulverizer. About 0.500 g smashed sample was accurately weighed into a 50 mL centrifugal tube. 20 mL methanol/water (60:40, v/v) was added in and the centrifugal tube was leave in an ultrasonic water bath to extraction for 30 min. Afterward the tube was put into a 60 °C water bath for 2 h and centrifuged at 3000 rpm for 5 min. The supernatant was filtered through a 0.22 μm filter membrane and collected as the sample solution. The standards solution was prepared by accurately weighed amount of 1.0 mg and dissolved with methanol in the 10 mL volumetric flask. All solutions were stored at 4 °C until analysis.

Chromatographic separation was operate with Shimadzu LC-20AD equipped with a Poroshell 120 EC-C18 column (100 × 4.6 mm, 2.7 μm). The mobile phase consists of acetonitrile (A) and water (B). Separation was achieved using the following linear gradient program: 0–10 min, 95%B–80%B; 10–15 min, 80%B–60%B; 15–25 min, 60%B; 25–50 min, 60%B–0%B; 50–60 min, 0%B; 60–60.1 min, 0%–95%B, 70 min, stop. The column temperature was maintained at 40 °C. A 10 μL aliquot of each sample was injected into the HPLC–UV system. The flow rate was set at 0.3 mL/min. The detection wavelengths were set at 203 nm, 270 nm and 325 nm. The five saponins concentration of the samples calculated with the chromatography of 203 nm.

### Similarity analysis with ALQFM

The fusion fingerprint of each *P. notoginseng* sample and their similarity were calculated with the software “Digitized Evaluation System for super-information Characteristics of TCM Chromatographic Fingerprints 4.0” (developed by Guoxiang Sun et al., Software certificated NO.0407573, China). The Average Linear Quantitative Fingerprint method (ALQFM) was adopted with the software to analyze the similarity^20,21^. There were three parameters involved. Average linear qualitative similarity (*Sm*), average linear quantitative similarity (*Pm*) and the fingerprint variation coefficient (*α*). The simplified parameter “Grade” was combined with *Sm*, *Pm* and *α* to evaluate the similarity of samples (Table 1). Generally, samples with the grade value ≤ 5 were recommended as the similar ones to the chosen reference sample.

**Table 1:** TCM similarity grades criteria based on ALQFM.

Grade	1	2	3	4	5	6	7	8
*Sm* ≥	0.95	0.9	0.85	0.8	0.7	0.6	0.5	*Sm* < 0.5
*Pm* ∈	95–105	90–110	80–120	75–125	70–130	60–140	50–150	0–∞
*α* ≤	0.05	0.10	0.15	0.20	0.30	0.40	0.50	α > 0.05

### Statistical analysis

Hierarchical clustering analysis (HCA) was performed according to the contents of the five saponins concentration of the 32 batch *P. notoginseng* samples to show the relatedness of the samples based on the composition indexes content. Heat map clustering and the principal component analysis (PCA) were performed according to the peak areas of 33 co-possessing peaks of the fusion fingerprints with the samples to show the relatedness of samples based on the fusion fingerprints. The co-possessing peaks’ areas of the fusion fingerprints with the *P. notoginseng* samples grouped according to their origin (Yunnan province or Sichuan province) were evaluated by Orthogonal Projection to Latent Structure Discriminant Analysis (OPLS-DA) to verify grouping and to find the peaks that can represent the difference between the two origins.

HCA was performed with the SPSS statistical system (version 13.0) using the between group linkage method with squared Euclidean distances. Heat map clustering were performed in R Programming Language and the similarity was measured with the between-groups linkage method and squared Euclidean. PCA and OPLS-DA were performed using SIMCA 14.1 software.

## Results and discussion

### Methodology evaluation of HPLC analysis

The calibration curves of five saponins standard solutions, as well as the linear ranges and the limit of detection (LOD) were showed in Supporting Information Table S1. All the standard products showed excellent linearity (R^2^ ≥ 0.999) over the tested concentration ranges. The quantitative accuracy was assessed with the standard addition method. The average recoveries for the five investigated compounds ranged from 91.84 to 105.38%. The fingerprint stability was evaluated with RSD values of each co-possessing fingerprint peak area were, respectively, less than 3.22%, 3.71% and 2.16% for the stability, precision and repeatability tests. Considering these results, the method was accurate and valid enough.

### Composition indexes content analysis

According to previous studies, saponins are the important functional components of *P. notoginseng*, and are often used as its indicator components. The sum of G-Rb_1_, G-Rg_1_ and NG-R_1_ content has been used as a marker for the quality control of *P. notoginseng* in Chinese Pharmacopoeia. In this study five saponins aboundant in *P. notoginseng* reported in our previous study^22^ are chosen as composition indexes. The content of five saponins contents in 32 *P. notoginseng* samples were showed in Table 2.

**Table 2:** The concentration of the five investigated compounds in the samples (mg/g).

Sample	G-Rb_1_	G-Rg_1_	G-Rd	NG-R_1_	NG-R_2_
S1	38.53	44.97	8.45	12.30	1.33
S2	39.00	51.62	7.63	11.09	0.87
S3	36.08	52.86	8.54	6.87	0.79
S4	25.92	42.00	5.11	4.64	0.49
S5	36.57	51.81	7.79	11.58	1.08
S6	30.76	39.28	6.79	5.95	0.61
S7	36.13	47.62	8.19	6.86	0.95
S8	35.44	35.40	7.37	6.96	0.57
S9	43.28	49.46	10.10	11.10	1.15
S10	31.00	39.92	7.02	8.82	0.79
S11	28.26	46.17	6.69	4.95	0.51
S12	42.40	46.75	11.42	11.91	1.38
S13	39.00	54.84	9.48	11.03	1.20
S14	37.22	44.20	9.23	7.96	0.98
S15	29.47	41.73	6.43	7.28	0.76
S16	32.17	45.56	6.46	7.75	0.93
S17	30.12	38.25	6.31	8.97	1.38
S18	20.47	29.86	3.53	6.30	0.87
S19	25.31	42.45	4.92	7.79	1.10
S20	22.49	25.41	5.04	8.08	0.91
S21	26.32	34.98	6.52	10.28	1.16
S22	31.28	30.11	6.57	8.01	1.09
S23	20.48	38.86	4.76	7.07	1.03
S24	22.90	35.51	6.19	8.02	1.23
S25	27.34	32.82	8.12	15.50	1.37
S26	25.79	36.53	8.25	6.06	0.85
S27	29.66	38.80	7.07	9.37	1.10
S28	23.30	39.43	6.29	10.10	1.04
S29	25.10	30.95	5.53	8.54	1.31
S30	24.25	34.25	6.28	7.31	1.20
S31	20.85	30.26	4.37	7.54	1.02
S32	20.08	29.34	3.81	7.86	1.17
Mean	29.91	40.06	6.88	8.56	1.01
RSD%	22.67	19.29	26.23	27.52	24.80

The HCA plot based on the five saponins contents is shown in Fig. S1. The failure of origin discrimination begins with the minimum between-groups distance. The result verified that it is impossible to distinguish the origin of *P. notoginseng* in Yunnan Province (the genuine producing area in plateau mountain area) and Sichuan Province (the new introduced producing area in hills in the hinterland of the basin) by the indicators content.

### HPLC fingerprint similarity analysis with ALQFM

**Figure 2:**
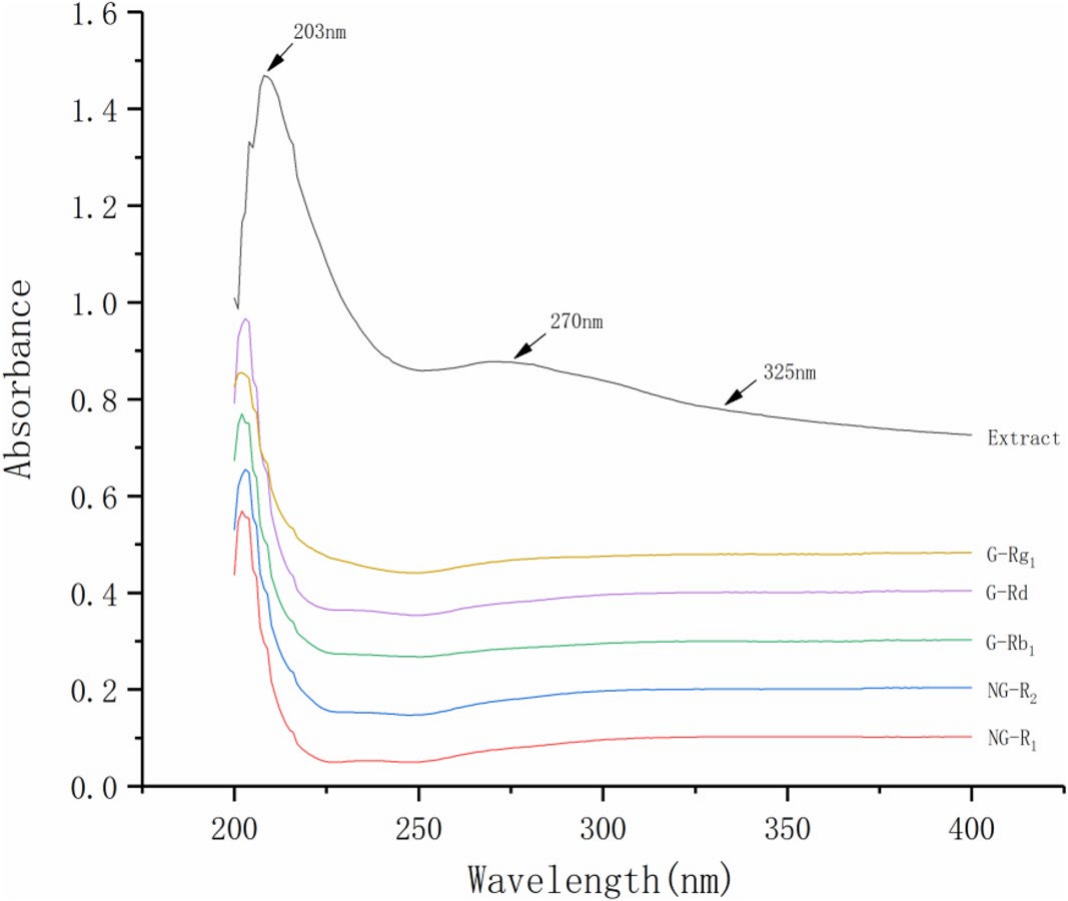
UV spectrum of five investigated saponins reference standards and *P. notoginseng* extract. *G-Rg*_*1*_ Ginsenoside Rg_1_, *G-Rd* Ginsenoside Rd, *G-Rb*_*1*_ Ginsenoside Rb_1_, *NG-R*_*2*_ Notoginsenoside R_2_, *NG-R*_*1*_ Notoginsenoside R_1_.

The UV spectra of the five saponins were investigated. The UV spectra of sample solution is different with that of the five saponins in 203–350 nm (shown in Fig. 2). In order to fully reflect the characteristics of the *P. notoginseng*, three absorption bands (203 nm, 270 nm and 325 nm) were chosen to establish the fusion fingerprint, which capable of synthesizing enhancing signal response and rich fingerprint information.

**Figure 3:**
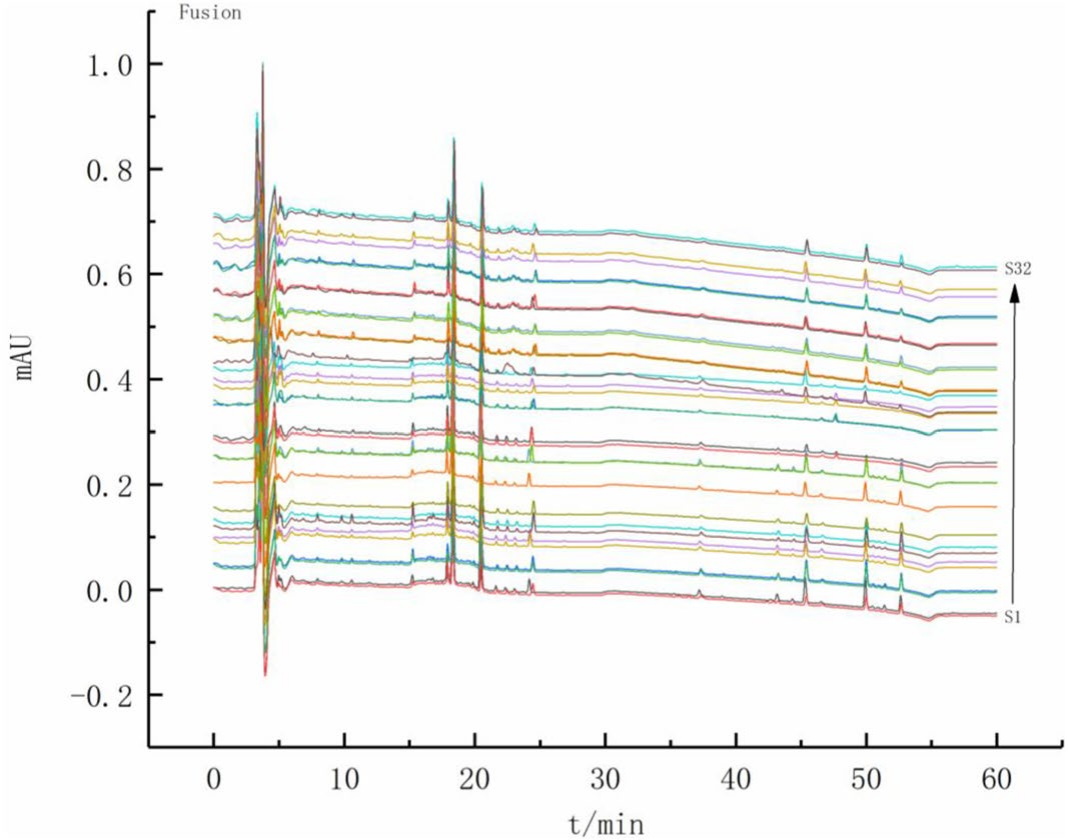
Fusion fingerprint of 32 batches of *P. notoginseng* samples. *S1* sample 1, *S32* sample 32.

The fingerprints of 32 sample solution with the three absorption bands (203 nm, 270 nm and 325 nm) were accomplished, respectively at first (shown in Supporting Information Fig. S2). Then the fusion fingerprint of each sample (shown in Fig. 3) and the similarity evaluation results (presented in Table 3) were calculated.

**Table 3:** The evaluation results obtained from ALQFM of 32 batches of *P. notoginseng* samples.

Sample	203 nm				270 nm				325 nm				Fusion			
	*Sm*	*Pm*%	α	Grade	*Sm*	*Pm*%	α	Grade	*Sm*	*Pm*%	α	Grade	*Sm*	*Pm*%	α	Grade
S1	0.962	117.4	0.031	4	0.929	116.5	0.009	4	0.93	123.6	0.01	5	0.883	94.3	0.047	3
S2	0.929	86.6	0.062	3	0.894	108.3	0.242	4	0.822	106.7	0.173	4	0.878	87.7	0.133	3
S3	0.951	106.6	0.017	2	0.927	111	0.027	3	0.963	138.5	0.009	6	0.936	113.2	0.007	3
S4	0.962	101.5	0.001	1	0.91	101	0.027	2	0.892	117.7	0.048	4	0.892	83.6	0.087	4
S5	0.988	109.4	0.004	2	0.971	111.3	0.07	3	0.955	112.4	0.031	3	0.899	86.4	0.09	3
S6	0.971	123.7	0.018	5	0.943	150.7	0.161	8	0.879	162.4	0.194	8	0.894	105.8	0.126	3
S7	0.974	122	0.004	5	0.953	138.8	0.164	6	0.874	151.5	0.18	8	0.893	101.3	0.111	3
S8	0.961	153.9	0.023	8	0.952	180.7	0.018	8	0.969	222.4	0.004	8	0.897	118.2	0.043	4
S9	0.953	91.5	0.015	2	0.93	88.9	0.095	3	0.965	80.6	0.033	4	0.892	78.9	0.119	5
S10	0.954	95.4	0.001	1	0.953	112.2	0.164	4	0.925	121.9	0.147	5	0.903	88.3	0.098	3
S11	0.96	114.2	0.023	3	0.936	127.2	0.015	5	0.938	138.4	0.043	6	0.901	94.2	0.064	2
S12	0.959	103.7	0.018	1	0.953	105.7	0.118	3	0.93	112.2	0.099	3	0.895	90.6	0.086	3
S13	0.961	114.4	0.03	3	0.964	121.6	0.016	5	0.939	155.2	0.074	8	0.897	82.9	0.075	4
S14	0.925	100.4	0.054	2	0.951	100	0.009	1	0.907	111.6	0.089	3	0.881	70.7	0.096	5
S15	0.933	102	0.077	2	0.974	99.3	0.032	1	0.917	100.1	0.027	2	0.888	72	0.136	5
S16	0.954	85.8	0.062	3	0.905	71.3	0.124	5	0.939	69.7	0.01	6	0.917	87	0.073	3
S17	0.948	78.7	0.057	5	0.901	67.4	0.117	6	0.912	64.9	0.072	6	0.913	79.5	0.069	5
S18	0.968	78.4	0.044	5	0.892	77.1	0.108	5	0.953	67.5	0.031	6	0.921	79.5	0.053	5
S19	0.978	84.1	0.003	4	0.947	77.8	0.119	5	0.91	69.3	0.077	6	0.922	86.1	0.011	3
S20	0.918	78.6	0.025	5	0.94	69.3	0.036	6	0.934	46.5	0.054	8	0.848	59.4	0.07	7
S21	0.963	100.4	0.068	2	0.962	83.1	0.024	4	0.957	65	0.027	6	0.882	69.3	0.127	6
S22	0.946	91.5	0.009	2	0.944	75.1	0.022	5	0.935	51.9	0.003	7	0.865	69.2	0.1	6
S23	0.932	85	0.078	4	0.931	69.9	0.017	6	0.911	58.4	0.016	7	0.864	60.6	0.158	6
S24	0.958	102	0.05	1	0.933	97.4	0.013	2	0.946	72.5	0.017	5	0.879	74.6	0.127	5
S25	0.953	91.9	0.001	2	0.965	87	0.03	3	0.947	75.8	0.037	5	0.873	70	0.083	5
S26	0.95	90.1	0.059	2	0.956	96	0.009	1	0.781	90.5	0.147	5	0.868	66	0.135	6
S27	0.957	94.3	0.018	2	0.968	78.5	0.034	5	0.961	70	0.005	6	0.881	71.2	0.104	5
S28	0.937	84.3	0.061	4	0.972	86.8	0.006	3	0.928	79.6	0.02	5	0.864	61.6	0.138	6
S29	0.957	92.2	0.052	2	0.968	76.4	0.01	5	0.945	59.6	0.021	7	0.878	66.2	0.126	6
S30	0.972	98.4	0.052	2	0.94	90.5	0.03	2	0.94	78.3	0.066	5	0.887	70.4	0.114	5
S31	0.957	92.3	0.061	2	0.964	91.1	0.012	2	0.947	75.8	0.023	5	0.892	64.4	0.111	6
S32	0.948	90.6	0.04	2	0.939	82.7	0.014	4	0.927	59.1	0.053	7	0.868	66.4	0.12	6

In ALQFM, qualitative analysis is performed first, then quantitative analysis is used. As Table 3 showed that all the samples had *α* < 0.30 and *Sm* > 0.90, indicating that the samples were similar in the distribution and number of chemical compositions. Based on Table 1, all the samples (*α* < 0.30, *Sm* > 0.90) should be in grade 1–5. Because of the difference content in the samples, some of the samples *Pm* < 70% or *Pm* > 130% which made their grades between 6 and 8.

If consider only qualitative similarity *Sm*, origin distinguish is not showed in any wavelength fingerprints nor in the fusion fingerprints. As further elaborated in follows, the addition of *Pm* makes the “Grade” have the function of origin discrimination, and the fusion fingerprints reflects an advantage in this respect.

### Correlation between *Grade* and origins

“Grade” is a simplified parameter for evaluating qualitative and quantitative similarity of samples of ALQFM method. The grades distribution of samples from different producing area were reflected with the scatter plots (Fig. S3). As shown in Fig. S3A–C the samples of the two producing areas cannot distinguish with grades distribution at any single wavelength, while as shown in Fig. S3D most of the samples (12 of 15) from Sichuan Province had a grade between 1 and 4, and most of the samples (15 of 17) from Yunnan Province had a grade between 5 and 8 calculated with fusion fingerprints. It can be seen that the fusion fingerprint is better than the single channel fingerprint in the respect of the producing areas distinguish capacity of Sichuan and Yunnan with *Grade*. This can be interpreted as the fusion fingerprints can reflect the sample characteristics more completely than the single-channel fingerprints because of the comprehensive utilization of the chromatographic information of the three bands of ultraviolet spectrum. The error frequency of using *Grade* to distinguish the origins is about 5 of 32.

### Relationship between fusion fingerprints and origins

**Figure 4:**
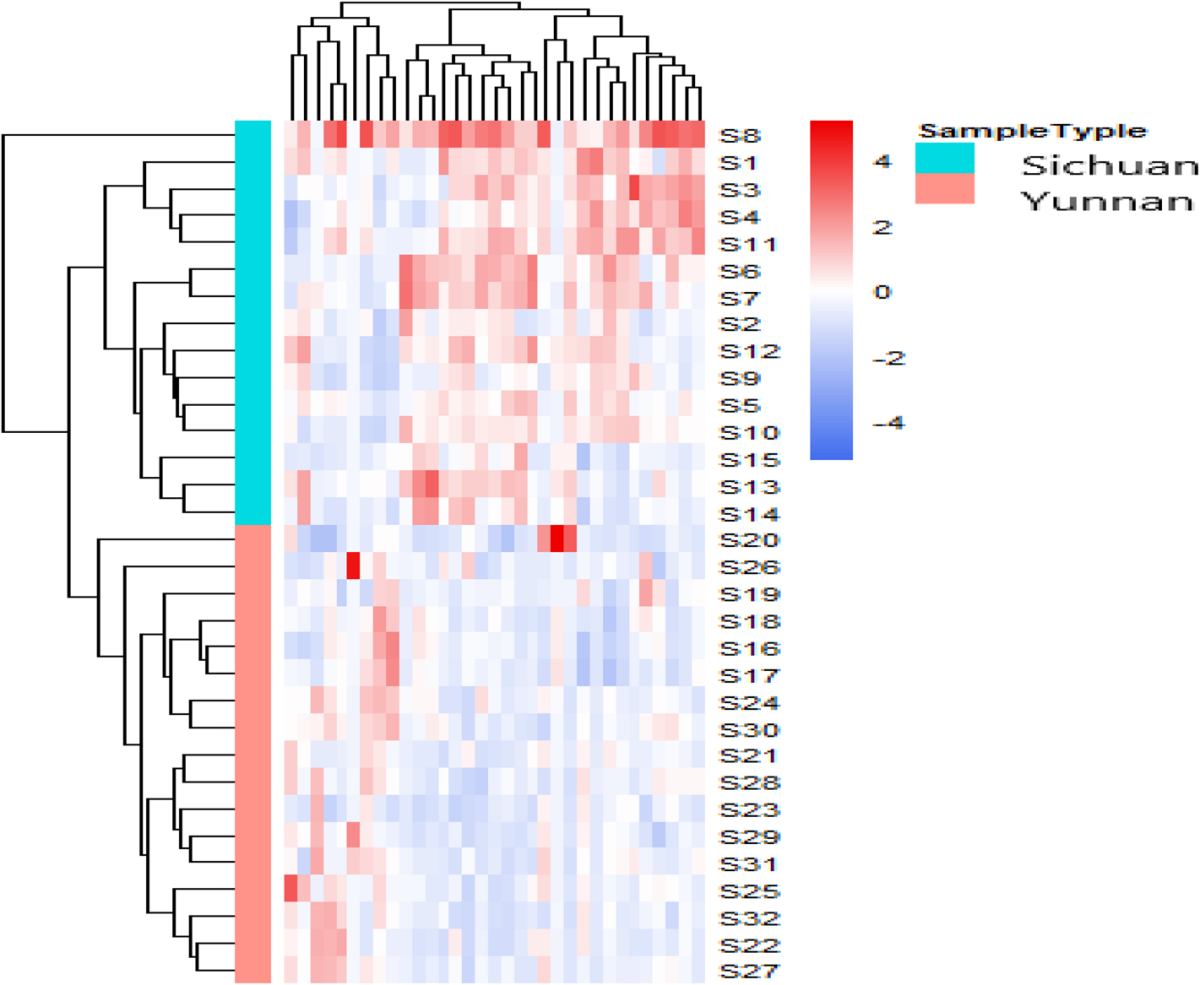
Hierarchical clustering analysis of 32 *P. notoginseng* samples from two producing area.

**Figure 5:**
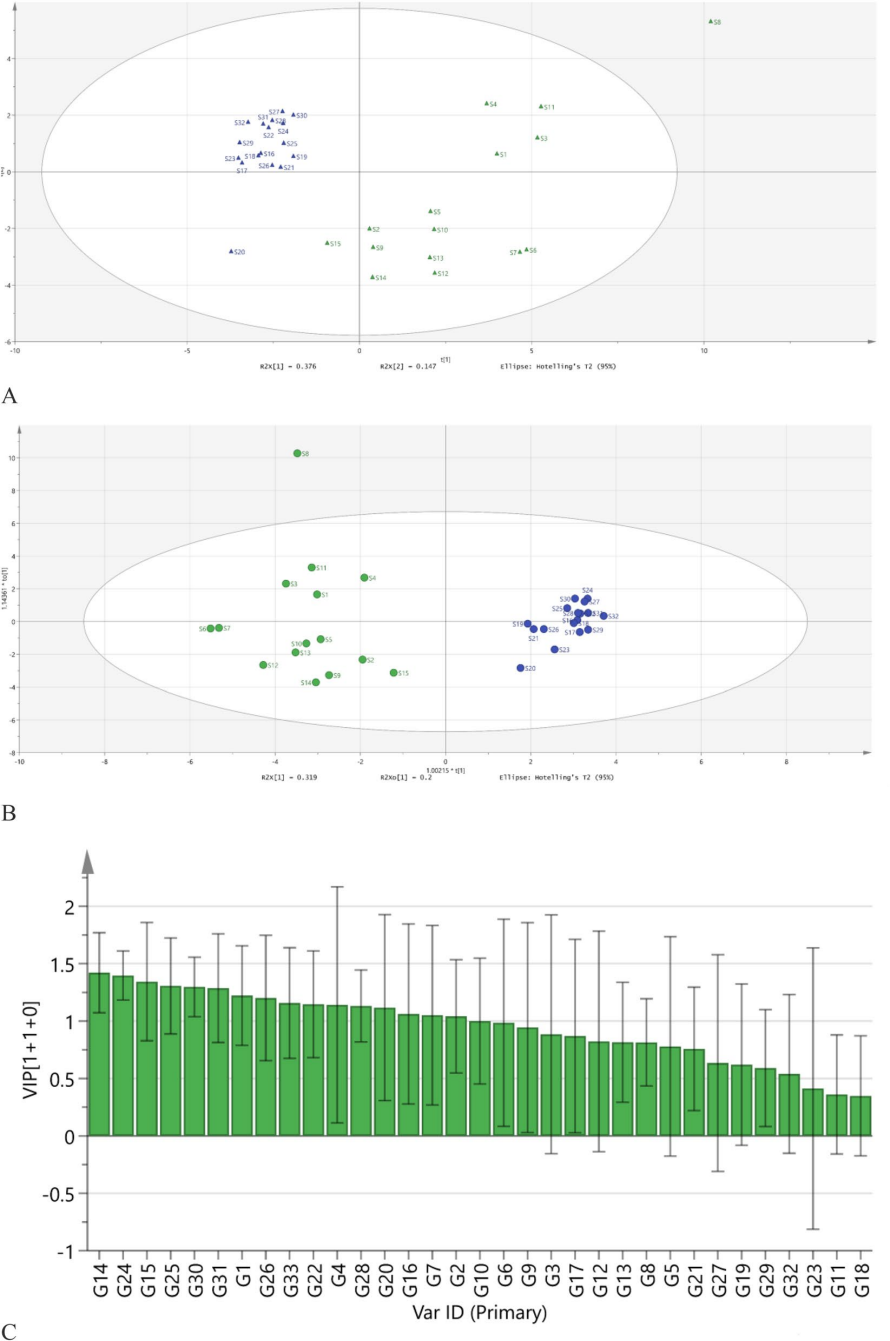
Principal component analysis score plots (**A**); orthogonal projection to latent structure discriminant analysis score plots (**B**); and VIP plots (**C**) for *P. notoginseng* samples.

The HCA heat-map (Fig. 4) shows the relatedness of 32 *P. notoginseng* samples based on the fusion fingerprints. Except S8, the samples are clustered into two groups, one group is S1–S15 and another group is S16–S32. The result is consistent with the origin distinguish (S1–S15 were collected from Sichuan province and S16–S32 were from Yunnan province). The PCA score plots (Fig. 5A) showed the classify result consistent with that of HCA heat-map.

That is to say, while the conventional unsupervised similarity evaluation analysis of the fusion fingerprints used to distinguish the origins between the two provinces the error frequency rate would reduce to 1 of 32.

To analyze the characteristic compounds most reflected to the difference of the *P. notoginseng* samples from the two provinces, Orthogonal Projection to Latent Structure Discriminant Analysis (OPLS-DA) were used to analyze the peaks which contributed to classification of the fusion fingerprints (Fig. 5B). Variable influence on projection statistics (VIP) values were calculated to reflect the contribution of each variable to the established model. The 15 peaks with VIP value > 1 in Fig. 5C were considered to be relevant for the classification significantly.

### Qualitative analysis of peaks relevant for the origin classification

High performance liquid chromatography combined with high resolution mass spectrometry (Orbitrap LC–MS, Thermo Scientific) was involved to analysis the sample. Data were processed with the software Exactive 2.8 with database searching (MZ Vault, Mass List, MZ Cloud). The results were shown in Table 4.

**Table 4:** Qualitative analysis of peaks relevant for the geographical origin classification.

Peak	Compounds	Molecular formula	Relative molecular mass (theory)	Feature fragment (m/z)	Mode	Retention time (min)
G14	DL-Tryptophan	C_11_H_12_N_2_O_2_	204.09	188.0707 [M + H-NH_3_]^+1^	ESI^+^	8.2
G24	Ginsenoside Rg_1_	C_42_H_72_O_14_	846.49	845.4900 [M −H]^−1^	ESI^−^	18.3
G15	Quercetin-3,4′-O-di-beta-glucopyranoside	C_27_H_30_O_17_	626.14	625.1419 [M − H]^−1^	ESI^−^	13.9
G25	Ginsenoside Rb_1_	C_54_H_92_O_23_	1109.29	1108.0963 [M − H]^−1^	ESI^−^	20.4
G30	Ginsenoside Rd	C_48_H_82_O_18_	947.15	946.0442 [M − H]^−1^	ESI^−^	24.0
G1	Proline	C_5_H_9_NO_2_	115.06	116.0709 [M + H]^+1^	ESI^+^	3.4
G26	Ginsenoside Rf	C_42_H_72_O_14_	800.49	845.4911 [M + FA-H]^−1^	ESI^−^	21.0
G28	Ginsenoside Rc	C_53_H_90_O_22_	1124.60	1123.5914 [M − H]^−1^	ESI^−^	22.5
G20	Rutin	C_27_H_30_O_16_	610.15	609.1464 [M − H]^−1^	ESI^−^	16.7

In Table 4 ginsenoside Rg_1_, ginsenoside Rb_1_, ginsenoside Rd are the selected compounds to investigate the concentration in this study, and the significant difference of their content in the *P. notoginseng* samples from two origins (P < 0.05) had been verified. The flavonoid and the amino acids identified as the characteristics compounds relevant with origin classification reflected the importance of non-saponin compounds in the quality control of the *P. notoginseng.*

The results in Table 4 are consistent with those in Table 2. In Table 2 there were significant differences in the G-Rb_1_, G-Rg_1_, G-Rd and NG-R_2_ content of the *P. notoginseng* samples from two producing areas and three of the four compounds with higher content were in Table 4. This result could be a verification of this geographical origin identification method.

## Conclusion

Compared with the single wavelength fingerprint, the fusion fingerprint has a significant advantage in geographical origin identification. This method comprehensive utilized of the chromatographic information of the three bands of ultraviolet spectrum, and has an advantage in the discrimination of geographical origin than single-channel fingerprints method. In this study, five saponins, two flavonoids and two amino acids showed significant correlation with the geographical origins. This suggested that in addition to the recognized active saponins, categories of active ingredients such as flavonoids and amino acids should also be paid attention to in the work of producing areas identification or the quality judgment of *P. notoginseng*.

## Supplementary Information

Supplementary Information.
