# Supplementary material for: Identification of geographical origins of Panax notoginseng based on HPLC multi-wavelength fusion profiling combined with average linear quantitative fingerprint method
Source: Sci Rep. 2021 Mar 4;11:5126. doi: 10.1038/s41598-021-84589-9 (PMC7933339; doi:10.1038/s41598-021-84589-9)
Supplement: Supplementary file 1 — Supplementary Information. [file 41598_2021_84589_MOESM1_ESM.pdf]

Identification of Geographical Origins of *Panax notoginseng* based on HPLC Multi-wavelength fusion profiling combined with Average Linear Quantitative Fingerprint method

Jing Bai, Pan Yue, Qiang Dong, Fang Wang, Chengyan He, Yang Li*, Jinlin Guo*

Chengdu University of Traditional Chinese Medicine, The Ministry of Education Key Laboratory of Standardization of Chinese Herbal Medicine, Key Laboratory of Systematic Research, Development and Utilization of Chinese Medicine Resources Key Laboratory Breeding Base, Chengdu 611137, China

Supplementary Information list

Table S1. Calibration curves and limit of detection (LOD) of five investigated compounds.

Fig. S1. The HCA plot of 32 batches of *P. notoginseng* samples based on the 5 saponins contents. S1-S32 are sample 1- sample 32.

Fig. S2. HPLC fingerprints of 32 batches of *P. notoginseng* samples at 203nm, 270nm and 325nm.

Fig. S3. Grades distribution of samples from different producing area.

Table S1. Calibration curves and limit of detection (LOD) of five investigated compounds.

Chemical compound	Regression equation	R^2	linear ranges（μg/mL）	limit of detection (LOD) （μg/mL）
G-Rb_1	y = 1514.3x - 3.6707	0.9998	10~200	2.00
G-Rg_1	y = 1828.8x + 65.455	0.9991	200~1400	0.16
G-Rd	y = 1561.1x - 9.3835	0.9990	50~500	0.32
NG-R_1	y = 1716.6x - 14.522	0.9990	100~1400	2.80
NG-R_2	y = 2561.7x - 4.1951	0.9992	10~200	0.18

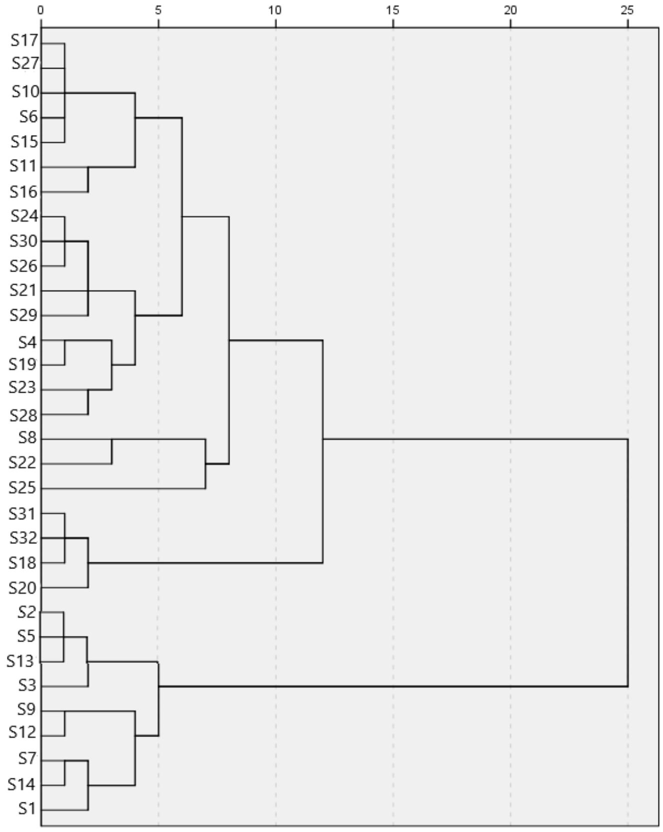

Fig. S1. The HCA plot of 32 batches of *P. notoginseng* samples based on the 5 saponins contents. S1-S32 are sample 1- sample 32.

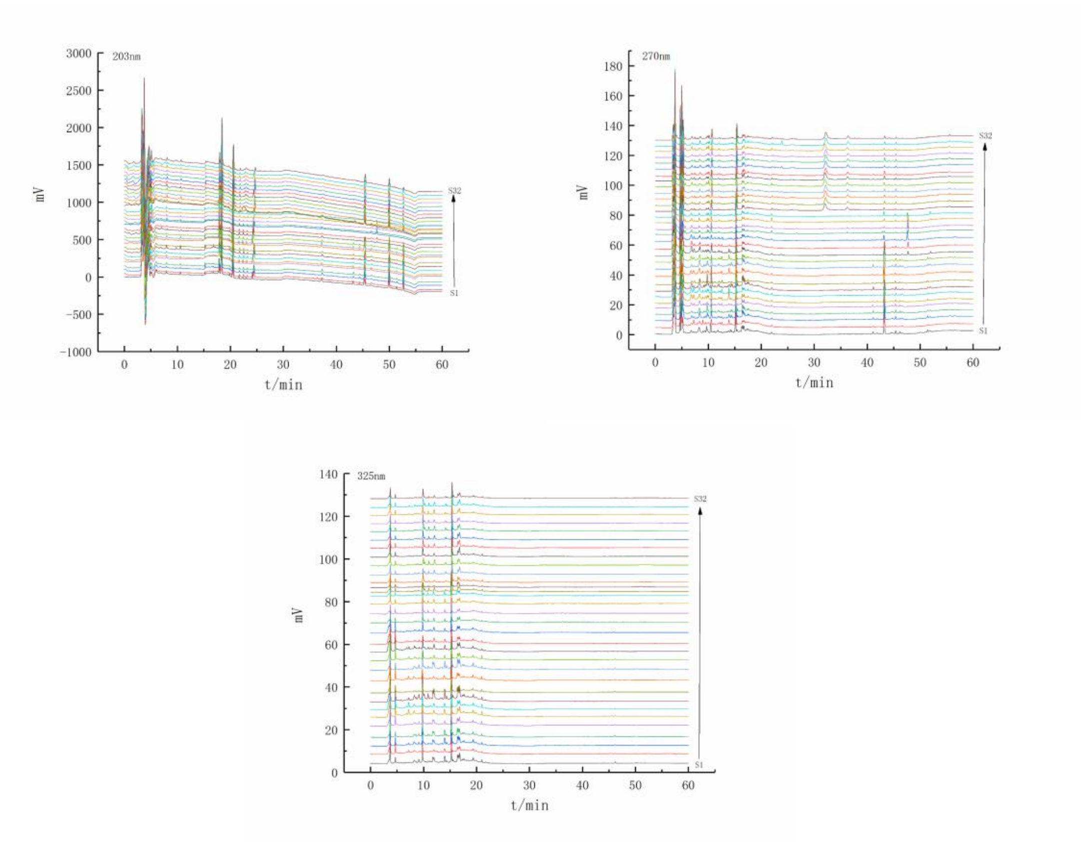

Fig. S2. HPLC fingerprints of 32 batches of *P. notoginseng* samples at 203nm, 270nm and 325nm.

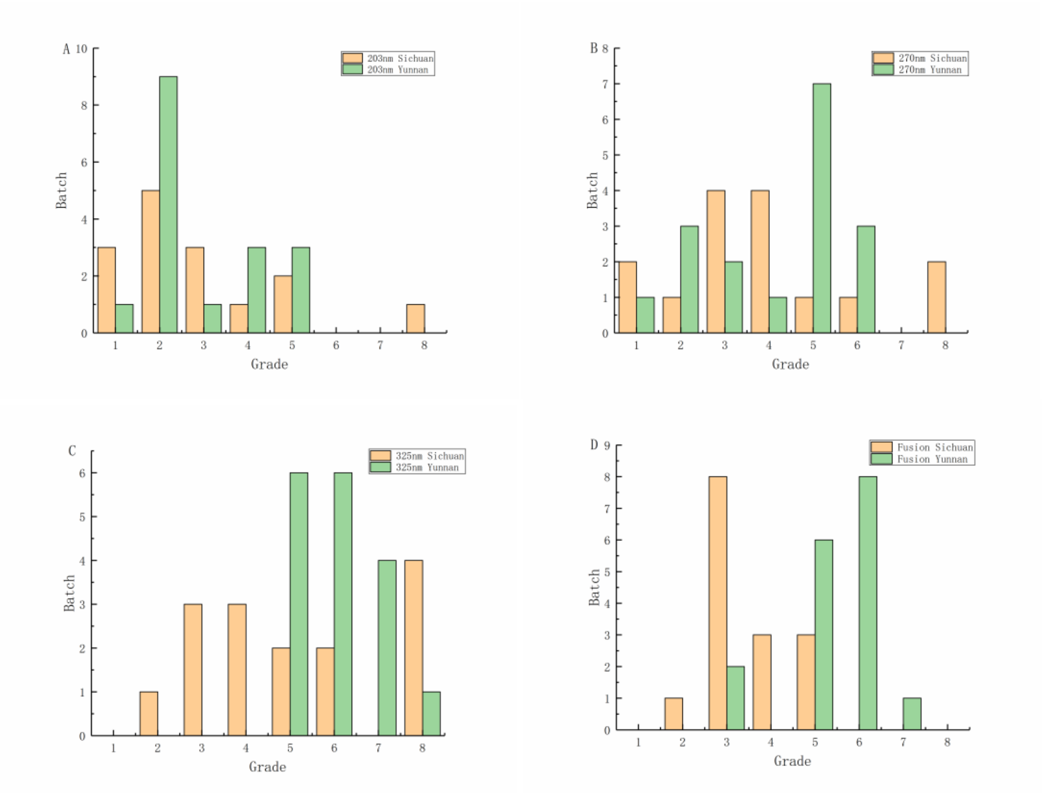

Fig. S3. Grades distribution of samples from different producing area.
